# Differences in the Distribution of Species, Carbapenemases, Sequence Types, Antimicrobial Heteroresistance and Mortality Rates Between Pediatric and Adult Carbapenemase-Producing *Enterobacterales* in Bloodstream Infections

**DOI:** 10.3389/fmed.2022.827474

**Published:** 2022-03-14

**Authors:** Hanbing Yu, Deyu Ma, Bo Liu, Suqing Yang, Qiuxia Lin, Renlin Yu, Xiaojiong Jia, Siqiang Niu, Qun Zhang, Shifeng Huang

**Affiliations:** ^1^Department of Laboratory Medicine, The First Affiliated Hospital of Chongqing Medical University, Chongqing, China; ^2^Department of Burn and Plastic Surgery, The First Affiliated Hospital of Chongqing Medical University, Chongqing, China; ^3^Chongqing Testing and lnspection Center for Medical Devices, Chongqing, China; ^4^Division of Allergy and Clinical Immunology, Brigham and Women's Hospital and Harvard Medical School, Boston, MA, United States; ^5^Department of Laboratory Medicine, The Affiliated Children's Hospital of Chongqing Medical University, Chongqing, China

**Keywords:** carbapenemase-producing *Enterobacterales*, bloodstream infections, antibiotic treatment, *bla*_NDM_, *bla*_KPC_, heteroresistance, combination therapy, children

## Abstract

The dissemination of carbapenemase-producing *Enterobacterales* (CPE) is worrisome given their scarce treatment options. CPE bloodstream infections (BSIs) had a high mortality rate in adults, and there was little data on pediatric CPE-BSIs around the world. We comprehensively explored the differences in the clinical and microbiological characteristics between pediatric and adult CPE-BSIs. Forty-eight pediatric and 78 adult CPE-BSIs cases were collected. All-cause 30 day-mortality in children with CPE-BSIs (14.6%, 7/48) was significantly lower than that in adult patients (42.3%, 33/78, *p* = 0.001). The subgroup in adults empirically treated with tigecycline as an active drug displayed a significantly higher 30-days crude mortality (63.3%, 19/30) than the subgroup treated without tigecycline (29.2%, 14/48, *p* = 0.003). *K. pneumoniae* was the most prevalent species in both the pediatric (45.8%, 22/48) and adult populations (64.1%, 50/78), with discrepant carbapenemase genes in each population: 95.4% (21/22) of the pediatric *K. pneumoniae* isolates carried *bla*_NDM_, while 82.0% (41/50) of the adult strains harbored *bla*_KPC_. The ratio of *E. coli* in children (37.5%) was significantly higher than that in adults (12.8%, *p* = 0.002). In both populations, the majority of *E. coli* expressed *bla*_NDM_, particularly *bla*_NDM−5_. With statistical significance, *bla*_NDM_ was much more common in children (95.8%, 46/48) than in adults (34.6%, 27/78). The rate of multiple-heteroresistance phenotypes in children was as high as 87.5%, which was much lower in adults (57.1%). Agar dilution checkboard experiment against one pediatric carbapenemase-producing *E. coli* isolates showed that the combination of amikacin and fosfomycin yielded an additive effect. Overall, *K. pneumoniae* was the most common CPE-BSIs pathogen in both populations, with NDM-producing *K. pneumoniae* and KPC-producing ST11 *K. pneumoniae* being the most prevalent species in children and adults, respectively. *E. coli* was more prevalent in children than in adults, yet *bla*_NDM−5_ was the most common carbapenem-resistant mechanism in *E. coli* in both populations. The wide range of multiple-heteroresistance combination traits found in different pathogen species from different host populations should provide a good foundation for future combination therapy design. Further investigations from more CPE isolates of various species are needed to evaluate the possible *in vitro* partial synergy of the amikacin and fosfomycin combination.

## Introduction

Carbapenemase-producing *Enterobacterales* (CPE), a predominant member of carbapenem-resistant *Enterobacterales* (CRE), pose a great threat to global health (1, 2). Bloodstream infections (BSIs) due to CPE (CPE-BSIs) tend to be persistent or recurrent; furthermore, the mortality of such infections can even reach 50% in children and 65% in adults (3–5). However, global CPE epidemics demonstrated important regional differences concerning patients' baseline characteristics, clinical outcomes, and bacterial characteristics. Research findings from one region might not be generalisable to other regions. In addition, compared to adults, little is known about the treatment strategies used by children with CPE-BSIs.

Various carbapenemases along with other resistance mechanisms contribute to the deleterious effect on CPE management. Typical carbapenemases include *Klebsiella pneumoniae* carbapenemase (KPC), Metallo-β-lactamases (MBLs), and Oxacillinase-48 (OXA-48), as representatives of Ambler class A, Ambler class B, and Ambler class D, respectively (6, 7). There are currently no active MBLs inhibitors in clinics, particularly for the subtype New-Delhi Metallo-lactamase (NDM) found in many *Enterobacterales* species (8, 9).

Timely active antibiotic treatment can improve the outcomes (10, 11), but there are few treatment options for CPE-BSIs. Antibiotic therapy now rely mostly on medications like colistin, tigecycline, fosfomycin, and aminoglycosides, which are all threatened by developing resistance and plagued by considerable side effects (12, 13). Moreover, both colistin and tigecycline are known as last-resort antibiotics used mainly to treat MDR infections. New antibiotics show superior bactericidal effects, for instance, ceftazidime-avibactam (CAZ-AVI) targeting KPC and OXA-48 producers (14), and aztreonam-avibactam (ATM-AVI) active against KPC, MBLs, AmpC, and OXA-48 producers (15), yet resistance to these drugs is still inevitable (16, 17). Combination therapy, pathogen virulence-targeting method, host immunity modulation strategy, and phage therapy are some of the most promising therapeutic strategies for combating antibacterial resistance (6, 18), among which combination therapy is strongly recommended to fight against highly resistant infections as it can not only slow down the acquisition of resistance but also improve bactericidal efficacy. Thus, efficient combination therapy is recommended to combat CPE-BSIs, especially in children for whom anti-infection treatments are mostly extrapolated from adults, and most last-resort drugs such as aminoglycosides are not recommended (19). Overall, a thorough understanding of the mechanistic principles governing antibacterial drug resistance is fundamental for the development of novel combination therapeutics to combat current and emerging bacterial threats. However, as most of the combination therapies were clinical experience-directed or observation-directed, with controversial results and few systematic experiments and clinical trials, combination therapy is still lacking solid ground (10). As drug combinations targeting multiple-heteroresistance mechanisms exhibited excellent activities (20), multiple-heteroresistance may be a critical foundation for antibiotic combination therapy. However, the effect of combination therapy is still sporadic with such a theoretical basis. Notably, combination therapy involves the drug repurposing and regrouping of the existing antimicrobial agents to provide a synergistic approach for management of infectious diseases by obtaining broad-spectrum coverage and minimizing resistance development, thus the rationale behind the choice of combination therapy is that the antimicrobials will have a synergistic effect when given together. Hence, it is not that any two or three antibiotics showing multiple-heteroresistance can be used in combination. Most medications are previously known to kill bacteria, and antimicrobials are generally classed as bacteriostatic or bactericidal agents. Combining a bacteriostatic (e.g., doxycycline) and a bactericidal (e.g., β-lactam) medicine might theoretically limit the growth required for the latter's mechanism of action. Therefore, one step in developing multiple-heteroresistance-based new combinations includes theoretical scrutiny and subsequent *in vitro* testing for synergy through the dilution checkerboard method.

Though dissemination of CPE in adults and related antibiotic treatments for CPE-BSIs are well-explored, there is still a knowledge gap in children with CPE-BSIs. Understanding the distinctions between children and adult CPE-BSIs would not only help to complete a full research of the mechanisms involved in CPE dissemination in children, but it will also help to enhance treatment options in both groups.

Here, we explored the differences between children and adults with CPE-BSIs mainly from two aspects: (1) clinical characteristics, and (2) microbiological characteristics. Besides, we investigated the heteroresistance phenotypes in all the CPE-BSIs isolates from both populations and evaluated the inspiration on the optimization of the treatment strategies by exploiting multiple-heteroresistance.

## Methods

### Patients and Strains

CRE is defined as *Enterobacterales* that are resistant to at least one carbapenem antibiotic (ertapenem, meropenem, doripenem, or imipenem) or produce a carbapenemase, according to the Centers for Disease Control and Prevention in the USA (https://www.cdc.gov/hai/organisms/cre/technical-info.html#Definition). Both adult and pediatric patients with mono-microbial CRE-BSIs from three tertiary teaching hospitals in the medical center of Chongqing Medical University from 2015 to 2020 were collected. Both the laboratory information system and the hospital information system were used to collect all relevant data, including demographics, underlying diseases, antimicrobial therapeutics, and microbiological data. Then the CRE-BSIs isolates carrying any of the tested carbapenemase genes (*bla*_KPC_, *bla*_NDM_, *bla*_VIM_, *bla*_IMP_, *bla*_OXA−48−*like*_) as confirmed by polymerase chain reaction (PCR) and DNA sequencing were recognized as CPE-BSIs strains, and patients with CPE-BSIs were included in this study.

Pediatric patients and corresponding strains were from the Affiliated Children's Hospital of Chongqing Medical University, while adult patients and related strains were from both the First Affiliated and the Second Affiliated Hospital of Chongqing Medical University. The above-mentioned three hospitals were large tertiary teaching hospitals from one medical center in Chongqing, China.

The VITEK MS (BioMerieux) was used to identify all CRE strains isolated from blood, which were then stored at −80°C in the laboratory.

### Detection of Carbapenemase-Encoding Genes and Extended-Spectrum β-Lactamases

Total DNA was extracted by the boiling centrifugation method. Briefly, single colonies were picked from an overnight culture of each isolate, resuspended in 200 ul of sterile distilled water, and boiled at 100°C for 10 min. After centrifugation at 15,000 × g for 15 min, supernatants were collected and stored at −20°C. The potential presence of both the carbapenemase genes (*bla*_KPC_, *bla*_NDM_, *bla*_VIM_, *bla*_IMP_, *bla*_OXA−48−*like*_) and the ESBLs genes (*bla*_SHV_, *bla*_TEM_, *bla*_CTXM_) was detected by PCR as described previously (21), and were further confirmed by DNA sequencing. The carbapenemase genes' primers were designed to amplify as many variants as possible. For example, the primers for *bla*_NDM_ covered *bla*_NDM−1_~*bla*_NDM−31_ and *bla*_NDM−34_~*bla*_NDM−41_; the primers for *bla*_KPC_ covered *bl*a_KPC−2_~*bla*_KPC−8_, *bla*_KPC−10_~*bla*_KPC−70_, *bla*_KPC−72_~*bla*_KPC−75_, *bla*_KPC−78_~*bla*_KPC−82_, *bla*_KPC−84_~*bla*_KPC−88_, *bla*_KPC−90_~*bla*_KPC−91_, *bla*_KPC−94_~*bla*_KPC−98_, *bla*_KPC−102_~*bla*_KPC−108_. Therefore, *bla*_NDM−1_~*bla*_NDM−31_, *bla*_NDM−34_~*bla*_NDM−41_, *bl*a_KPC−2_~*bla*_KPC−8_, *bla*_KPC−10_~*bla*_KPC−70_, *bla*_KPC−72_~*bla*_KPC−75_, *bla*_KPC−78_~*bla*_KPC−82_, *bla*_KPC−84_~*bla*_KPC−88_, *bla*_KPC−90_~*bla*_KPC−91_, *bla*_KPC−94_~*bla*_KPC−98_, and *bla*_KPC−102_~*bla*_KPC−108_ were screened.

### Multi-Locus Sequence Typing

MLST analyses were performed on *Klebsiella pneumoniae, Escherichia coli, Enterobacter cloacae* and *Citrobacter freudii*, according to the protocols established by BIGSdb-Pasteur (https://bigsdb.pasteur.fr/index.html) and PubMLST (https://pubmlst.org/).

### Broth Microdilution Methods

Minimum inhibitory concentrations (MICs) of ertapenem, meropenem, imipenem, ceftazidime, ceftazidime-avibactam, aztreonam, aztreonam-avibactam, polymyxin B, and tigecycline were established by broth microdilution methods recommended by the Clinical and Laboratory Standards Institute (CLSI) (22). *E. coli* ATCC25922 was employed as the control strain. The MIC breakpoints of tigecycline and polymyxin B were from EUCAST (23), and the others were from CLSI.

### Disk Diffusion Methods

According to CLSI (22), overnight cultures on Columbia blood agar were suspended in 0.9% saline to achieve 0.5 McFarland units (~1.5 × 10^8^ CFU/ml), then the inoculums were spread onto Mueller-Hinton (MH) plates by sterile cotton swabs, and disks were applied by tweezers or dispensers. The category of the disks (Oxoid) used was as follows: ceftriaxone (CRO, 30 μg), cefepime (FEP, 30 μg), ceftazidime (CAZ, 30 μg), piperacillin/tazobactam (TZP, 100 ug/10 μg), ceftazidime-avibactam (CAZ-AVI, 30 μg/20 μg), aztreonam (ATM, 30 μg), ertapenem (ETP, 10 μg), imipenem (IPM, 10 μg), meropenem (MEM, 10 μg), tobramycin (TOB, 10 μg), amikacin (AK, 30 μg), tetracycline (TE, 30 μg), minocycline (MH, 30 μg), ciprofloxacin (CIP, 5 μg), fosfomycin (FOS, 200 μg), tigecycline (TGC, 15 μg), polymyxin B (PB, 300 μg), colistin (CT, 10 μg). Plates were then incubated at 37°C for 48 h, during which time the diameters of the inhibition zones and heteroresistance phenotypes were measured and recorded.

Dual disks diffusion tests were carried out according to the same protocol, whereas the distance of the two disks placed was half of the sum of diameters of the inhibition zones of the two disks.

### Population Analysis Profile

PAP analyses were conducted as described previously (24). Two or three colonies from the overnight cultures on Columbia blood agar were suspended in 1 ml MH broth respectively, and incubated overnight at 37°C. After that, each culture was diluted 1:1000 in PBS buffer, and 1 μl of each was inoculated into a vial with 1 ml of MH broth. After overnight growth, 10^−1^ to 10^−4^ dilutions in PBS buffer were prepared. Five microlitre from each suspension (0~10^−4^) was spread onto the freshly prepared MH agar plates with or without increasing amounts of antibiotics (FOS and AK). The concentration of antibiotics was from ¼ × MIC to 8 × MIC (2 fold increments). All plates were incubated overnight at 37°C and colonies were counted to determine the frequency of bacteria growing at each antibiotic concentration. Strains with colonies which appeared on 4 × MIC or 8 × MIC plates at a frequency of 10^−7^ or higher were recognized as heteroresistant strains (20).

### E-Test

Overnight cultures on Columbia blood agar were suspended in 0.9% saline to achieve 0.5 McFarland units (~1.5 × 10^8^ CFU/ml), and then the inoculum was spread onto MH plates by sterile cotton swabs, and equivalent fosfomycin (including 25 μg/ml G-6-P) and amikacin MIC Test Strips (Liofilchem) were applied by tweezers. Plates were subsequently incubated for 24 h at 37°C, and MICs were read after 24 h and 48 h. Synergy tests were carried out according to the protocol of the MTS^TM^ Synergy Applicator System of Liofilchem (25).

### Agar Dilution Checkboard Assay

A series of agar plates (containing 25 mg/L G-6-P) with or without mixed antibiotics (fosfomycin and amikacin) were prepared in the form of 8 × 8 matrix, and the concentrations of FOS from right to left and AK from top to bottom ranged from 1/16 × MIC to 4 × MIC (2-fold increments). Overnight cultures at 0.5 McFarland units were diluted 1:10 in 0.9% saline, and then 2 μl of the suspensions were inoculated onto the plates. All plates were incubated at 37°C for 16–20 h. New MICs of each isolate to each drug alone or drug combinations were determined. The Fraction Inhibitory Concentration Index (FICI) values were calculated according to the following formula: FICI = (MIC _drug A with drug B_/MIC _drug A alone_) + (MIC _drug B with drug A_/MIC _drug B alone_). The FICI results were interpreted as synergistic (≤ 0.5), additive (>0.5 to ≤ 1), indifferent (>1 to <4) or antagonism (≥ 4).

### Statistical Analysis

The age of adults was presented as average, and the rest variables in the study were presented as absolute frequencies or percentages. The clinic-related categorical variables in both populations were compared using χ^2^ test or Fisher's exact test, as appropriate. The percentages of various species distribution and resistance mechanisms in both populations were compared by Fisher's exact test. The Kaplan-Meier analysis was performed for the survival analysis. A two-tailed *P*-value of ≤ 0.05 was considered statistically significant. All statistical analysis was finished by SPSS25.0 (IBM).

## Results

### Clinical Characteristics of Pediatric and Adult Patients With CPE-BSIs

Baseline information (Table 1) 48 CRE isolates carrying any of the tested carbapenemase genes were identified as CPE among 52 cases of children with CRE-BSIs collected, and 48 cases of pediatric patients with CPE-BSIs were thus included in the study. Forty-two (87.5%) pediatric patients with CPE-BSIs were under 1 year old (neonates), and 30 (62.5%) children were male. Meanwhile, 39 (81.3%) pediatric patients were with nosocomial infections.

**Table 1:** Clinical characteristics of children and adults with CPE-BSIs.

**Characteristics**	**Children** ***N* = 48** ***n* (%)**	**Adults** ***N* = 78** ***n* (%)**	***P***
**Demographics**			
Age			
<1 month	22 (45.8)	-	
1 month−1 year	20 (41.7)	-	
1−2 year	4 (8.3)	-	
3−6 year	1 (2.1)	-	
>6 year	1 (2.1)	-	
18–25	-	3 (3.8)	
26–35	-	7 (9.0)	
36–50	-	15 (19.2)	
51–70	-	30 (38.5)	
71–90	-	23 (29.5)	
Male	30 (62.5)	55 (70.5)	0.434
Nosocomial infection	39 (81.3)	61 (78.2)	0.822
ICU acquired	17 (35.4)	23 (29.5)	0.556
**Underlying disease**			
Premature	11 (22.9)	-	
Cardiovascular disease	34 (70.8)	33 (41.5)	**0.003**
Blood disease	28 (58.3)	16 (20.5)	**0.000**
Gastrointestinal disease	22 (45.8)	45 (57.7)	0.205
Gastrointestinal bleeding	11 (22.9)	7 (9.0)	**0.038**
Respiratory disease	40 (83.3)	28 (35.9)	**0.000**
Respiratory failure	21 (43.8)	12 (15.4)	**0.001**
**Symptoms**			
Septic shock	6 (12.5)	8 (10.3)	0.773
Hypoproteinemia	34 (70.8)	50 (64.1)	0.560
Anemia	3 (6.3)	12 (15.4)	0.161
**Other infections**			
Pulmonary infection	42 (87.5)	37 (48.9)	**0.000**
Abdominal infection	12 (25.0)	13 (16.7)	0.358
Intracranial infection	6 (12.5)	2 (2.6)	0.053
Urinary tract infection	2 (4.2)	16 (20.5)	**0.016**
Tissue infection	4 (8.3)	5 (6.4)	0.730
**Treatment**			
Transfusion history	29 (60.4)	38 (48.7)	0.270
Glucocorticoids treatment	18 (37.5)	10 (12.8)	**0.002**
Arteriovenous indwelling tube	35 (72.9)	61 (78.2)	0.524
Tracheal intubation	35 (72.9)	34 (43.6)	**0.002**
Drainage tube	19 (39.6)	50 (64.1)	**0.010**
Urinary catheter	16 (33.3)	56 (71.8)	**0.000**
Mechanical ventilation	28 (58.3)	31 (39.7)	**0.046**
Stomach tube	21 (43.8)	31 (39.7)	0.711
Surgery within a month	17 (35.4)	42 (52.6)	0.063

*Note: Bold face indicates values that are statistically significant (P ≤ 0.05)*.

In the meantime, the study included 78 adult patients with CPE-BSIs. The mean age of the patients was 58 years old, with a minimum age of 18 and a maximum age of 88. Fifty-five (70.5%) were male, and 61 (78.2%) were with hospital-acquired infections.

**Figure 1:**
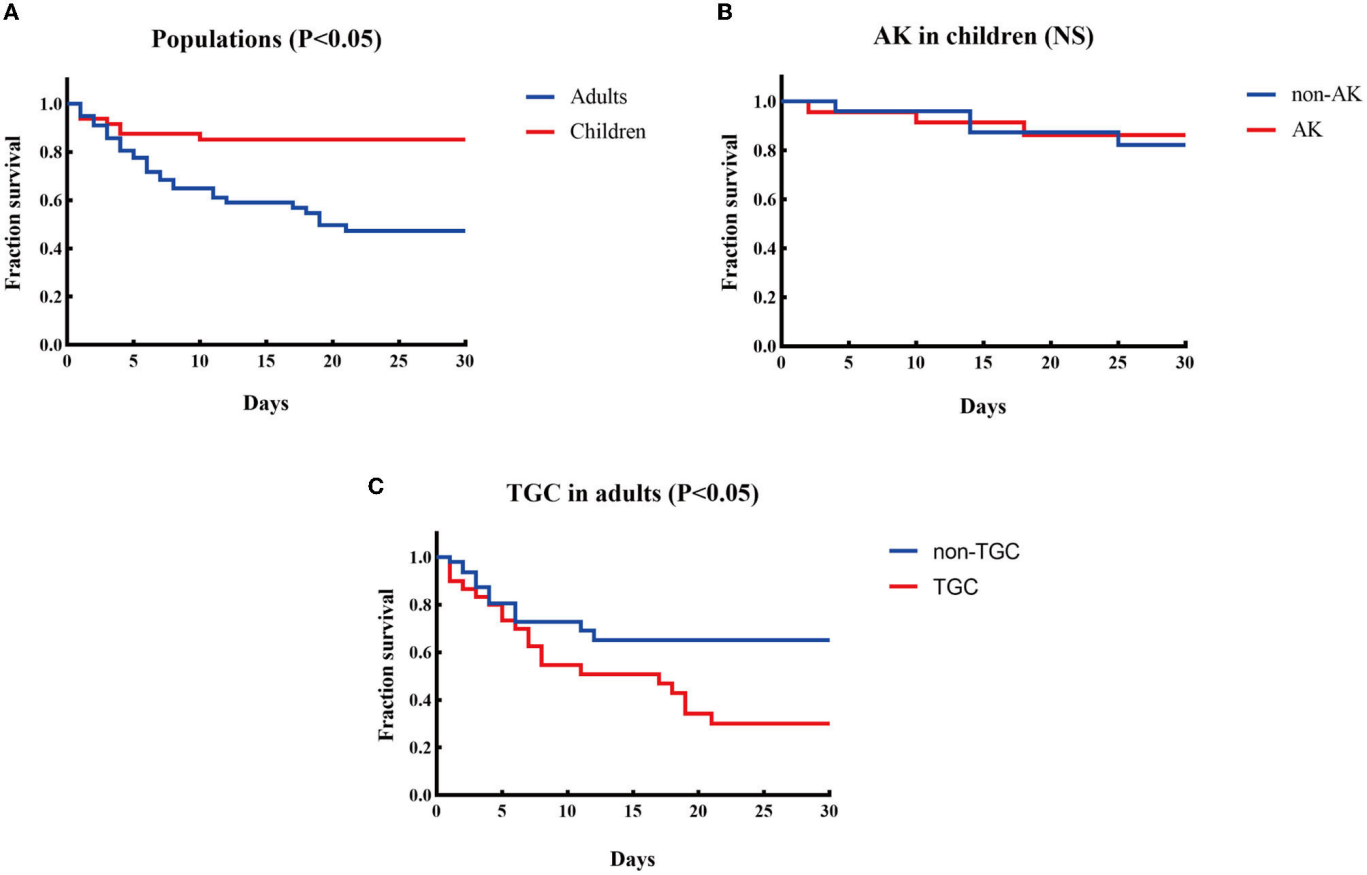
Kaplan-Meier curves of different factors and survival at 30 days in patients with CPE-BSIs. **(A)** The difference of 30-day crude mortality between adults and children. **(B)** The difference in crude mortality after 30 days between children who received AK as an active drug empirically and those who did not. AK, amikacin. **(C)** The difference of 30-day crude mortality between the adults who received TGC as an active drug empirically and the adults who didn't. TGC, tigecycline; NS, non-significant.

**Treatment and outcomes:** All-cause mortality within 30 days from the onset of CPE-BSIs of children was 14.6% (7/48), which was significantly lower than that of the adults (42.3%, 33/78, *p* = 0.001) (Figure 1A).

Amikacin was the most commonly prescribed drug in children's treatment (45.8%, 22/48), with 19 monotherapy and 3 combination therapy. Besides, 22 children received amikacin as the active drug. There was no significant difference in the unadjusted 30-day-crude mortality between the subgroup who received amikacin and the subgroup who did not (Figure 1B).

On the contrary, tigecycline was the most frequently prescribed drug in adults (38.5%, 30/78), and tigecycline was active in all the corresponding strains *in vitro*. The mainstay of combination therapies was tigecycline, but the other drug was not always fixed. Surprisingly, the subgroup given tigecycline as an active drug had a higher 30-day crude mortality rate (63.3%, 19/30) than the subgroup given no tigecycline (29.2%, 14/48, *P* = 0.003) (Figure 1C).

### Microbiological Characteristics of Pediatric and Adult Patients With CPE-BSIs

**Figure 2:**
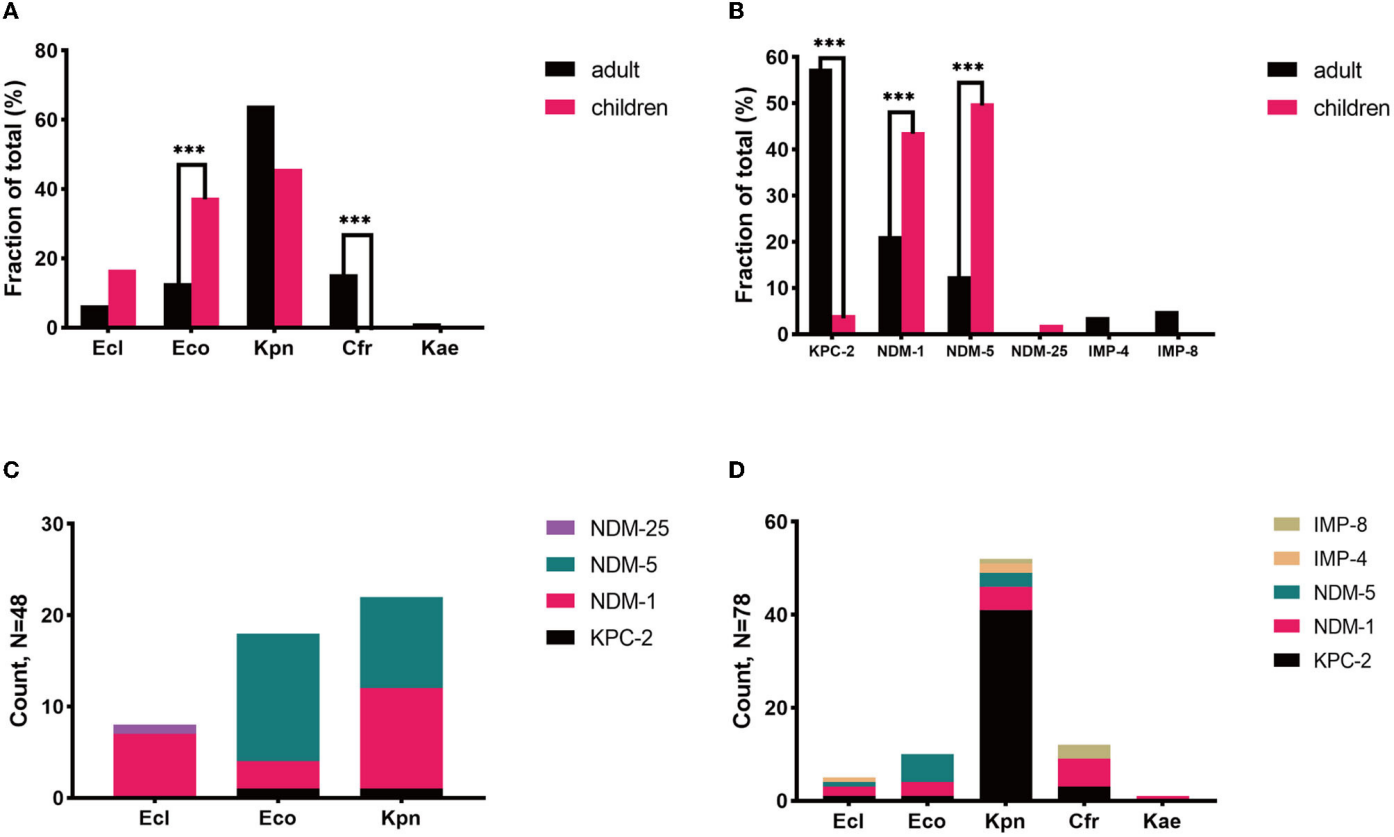
Microbiological characteristics of pediatric and adult CPE-BSIs patients. **(A)** The differences of species distribution in both populations. **(B)** The differences of carbapenemase-encoding genes distribution in both populations. **(C)** The carbapenemase-encoding genes distribution in the species from pediatric patients. **(D)** The carbapenemase-encoding genes distribution in the species from adult patients. ***P < 0.05. Kpn, *K. pneumoniae*; Eco, *E. coli*; Ecl, *E. cloacae*; Cfr, *C. freudii*; Kae, *Klebsiella aerogenes*.

*K. pneumoniae* was the most prevalent species in both populations, with discrepant carbapenemase genes in each population. *K. pneumoniae* was discovered to be the most common species in both patient groups, accounting for 45.8% (22/48) and 64.1% (50/78) of CPE-BSI isolates in the pediatric and adult populations, respectively, nevertheless with discrepant carbapenemase genes in each population: In children, 95.4% (21/22) of the *K. pneumoniae* isolates carried *bla*_NDM_, with 11 being *bla*_NDM−1_ and the other 10 being *bla*_NDM−5_; By contrast, 82.0% (41/50) of the *K. pneumoniae* strains in adults carried *bla*_KPC_, with all being subtype *bla*_KPC−2_ (Figures 2C,D).

Besides, most *E. cloacae* isolates in both populations carried MBLs genes, and most *C. freudii* strains, exclusively identified in adults, also carried MBLs.

Of note, ESBLs were prevalent in both populations. 93.7% of the CPE isolates in children co-expressed ESBLs, while 84.6% of the isolates in adults co-expressed ESBLs.

*E. coli* and *bla*_NDM_ were of high proportion in children. *While K. pneumoniae* was the most prevalent species in both populations, *E. cloacae* were found in both pediatric and adult populations, yet *C. freudii* and *Enterobacter aerogenes* were only identified in adults. Notably, the proportion of *E. coli* in children (37.5%) was significantly higher than that in adults with CPE-BSIs (12.8%, *p* = 0.002) (Figure 2A).

While *bla*_KPC−2_ (46/78, 59.0%) was the most common carbapenemase-encoding gene in adults, *bl*a_NDM_, including *bl*a_NDM−5_ (24/48. 50.0%), *bl*a_NDM−1_ (21/48, 43.8%), and *bl*a_NDM−25_ (1/48, 2.1%), was the predominant one in children (46/48, 95.8%). Furthermore, *bl*a_NDM_ was much more prevalent in children (95.8%, 46/48) than in adults (34.6%, 27/78) with statistical significance (*p* < 0.001) (Figure 2B).

Most *E. coli* expressed *bla*_NDM_ in both populations, especially *bla*_NDM−5_. In children, 94.4% (17/18) of the *E. coli* isolates carried *bla*_NDM_, with 82.4% (14/17) of *bla*_NDM_ being the subtype *bla*_NDM−5_. In adults, 90.0% (9/10) of the *E coli* strains carried *bla*_NDM_, and 66.7% (6/9) of *bla*_NDM_ was the *bla*_NDM−5_ subtype (Figures 2C,D).

### MLST in Strains With *bla*_NDM_ Was Complex

**Figure 3:**
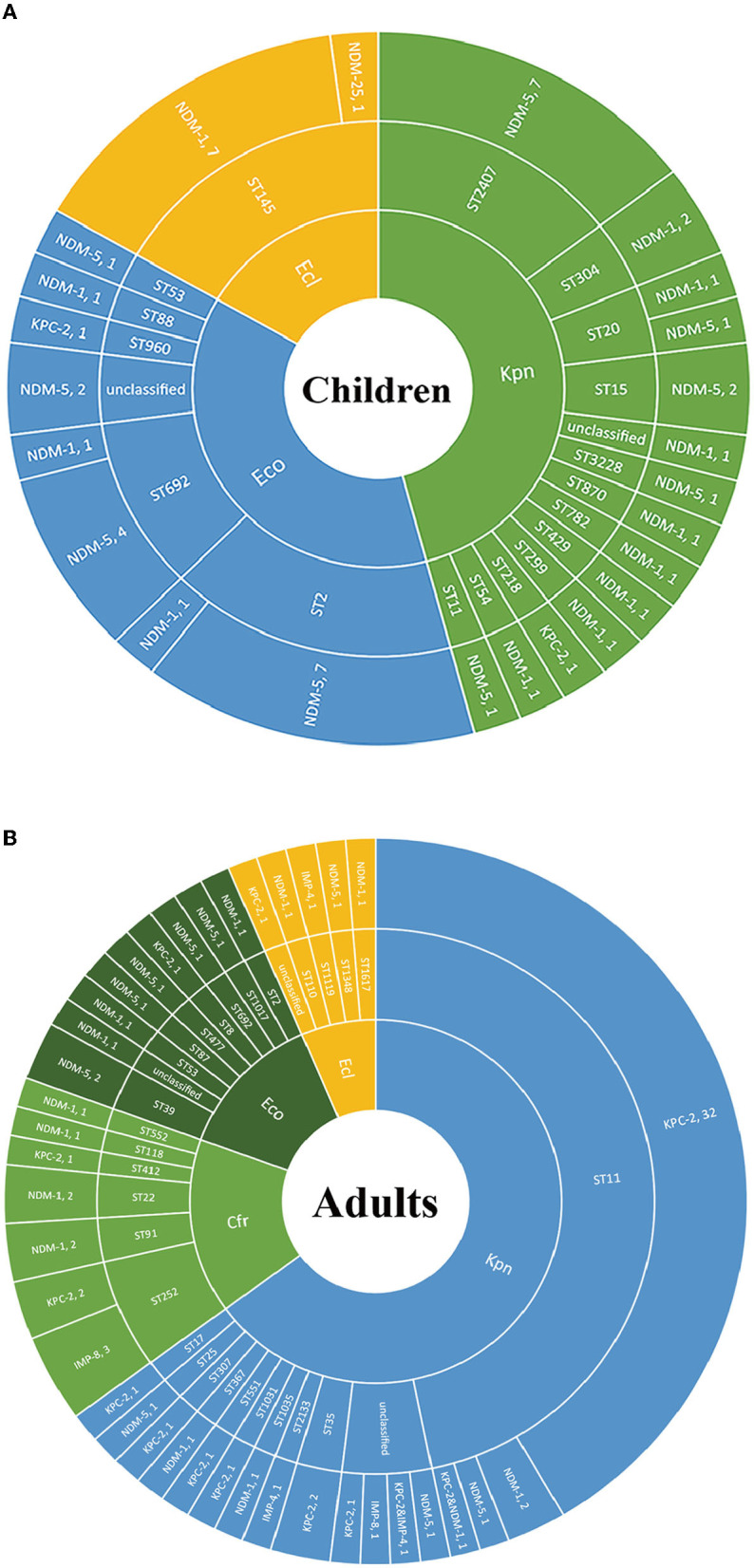
The Sunburst chart of the relationships among populations, species, STs, and carbapenemase distributions. The smallest circle indicates the species. The second-largest circle indicates the STs distributions in different species. The largest circle indicates the carbapenemase distributions in different STs in diverse species. The text before the comma indicates the carbapenemase's name, and the number after the comma indicates the number of the isolates with the defined carbapenemase, vice versa. **(A)** Children. **(B)** Adults. Kpn, *K. pneumoniae*; Eco, *E. coli*; Ecl, *E. cloacae*; Cfr, *C. freudii*.

We further explored the possible roles sequence types (STs) of different species played during the dissemination of CPE in both populations. As shown in Figure 3, regardless of species, various STs were demonstrated to carry *bla*_NDM_. In children, 7 out of 8 ST2 *E. coli* and 4 out of 5 ST692 *E. coli* expressed *bla*_NDM−5_, while there were no superior STs in *E. coli* isolates harboring *bla*_NDM_ in adults. In *E. cloacae*, similar findings have been found. Seven out of 8 ST145 *E. cloacae* isolates in children carried *bla*_NDM−1_, whilst no superior ST was found in *E. cloacae* isolates in adults. The MLSTs of *bla*_NDM_-harboring *K. pneumoniae* isolates in children were also varied, with no ST showing evident superiority. While 33 out of the 41 (80.5%) *bla*_KPC−2_-positive *K. pneumoniae* isolates in adults belonged to ST11, 7 out of the 21 (33.3%) pediatric *bla*_NDM_-carrying *K. pneumoniae* strains belonged to ST2407.

### ATM-AVI Exhibited Excellent *in vitro* Antibacterial Activity Against CPE-BSIs Isolates From Both Populations

Most of the isolates in both populations were resistant to all the three carbapenem antibiotics, including IPM, MEM, and ETP. Of note, the resistance rate of MEM was the lowest among the three. While none of the adult CPE-BSIs isolates were tigecycline-resistant, 12.5% of the pediatric strains were resistant to tigecycline. CPE-BSI isolates from children, on the other hand, had a much lower polymyxin B resistance rate (4.2%) than those from adults (14.3%). The susceptibility rates of ATM-AVI were 100.0% in children and 98.7% in adults, with the non-susceptible adult isolate being intermediate resistant. As for CAZ-AVI, while 85.4% of the pediatric isolates showed resistance, only 37.7% of isolates from adults were resistant (Table 2).

**Table 2:** MICs (mg/L) of the classical antibiotics against CPE-BSI isolates from children and adults.

**Antibiotic agents**	**All CPE-BSIs strains (Adults, *N* = 78)**				**All CPE-BSIs strains (Children, *N* = 48)**			
	**Range (mg/L)**	**S%**	**I%**	**R%**	**Range (mg/L)**	**S%**	**I%**	**R%**
Imipenem	≤ 1–256	6.4	1.3	92.3	<1–256	8.3	6.3	85.4
Meropenem	≤ 1–256	12.8	9.0	78.2	<1–64	16.7	8.3	75.0
Ertapenem	≤ 1–256	0.0	3.8	96.2	2–256	0.0	0.0	100.0
Aztreonam-avibactam	≤ 0.0625–8	98.7	1.3	0.0	<0.125–8	100.0	0.0	0.0
Aztreonam	≤ 1–≥ 64	16.7	0.0	83.3	<1–256	14.5	4.2	81.3
Ceftazidime	0.25–≥ 64	2.6	0.0	97.4	16–> 256	0.0	0.0	100.0
Ceftazidime-avibactam	0.25–>256^*^	62.3	–	37.7	<0.125–> 256	14.6	–	85.4
Tigecycline	<0.125–2^*^	94.8	–	0.0	<0.125–4	85.4	–	12.5
Polymyxin B	1–64^*^	85.7	–	14.3	1–4	95.8	–	4.2

* *: 77 of 78 stains from adults were tested, and the rest one was unavailable. -: There are no intermediate resistance breakpoints for the drugs*.

### Common Multiple-Heteroresistance Phenotype: A Possible Way to Design the Combination Therapy Strategy

We next explored the antimicrobial heteroresistance phenotypes in CPE-BSIs isolates to find the possible ways to design a combination therapy strategy.

**Figure 4:**
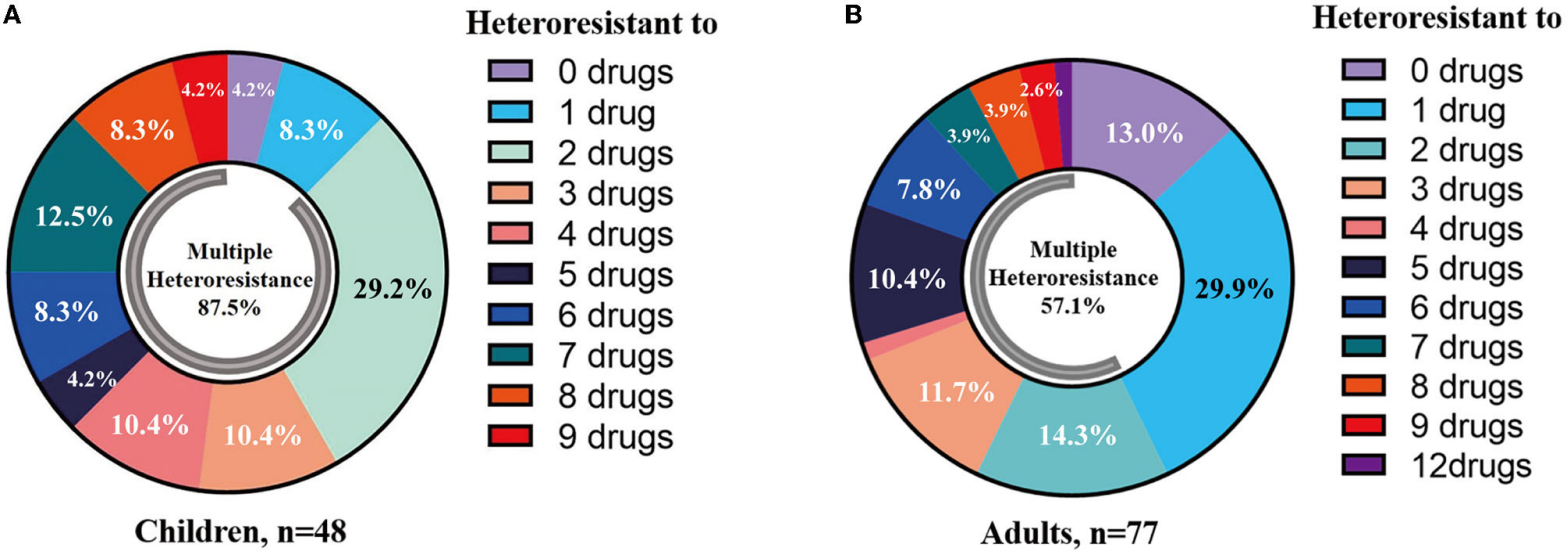
Multiple-heteroresistance phenotypes were common in both populations. **(A)** Multiple-heteroresistance phenotypes in children. **(B)** Multiple-heteroresistance phenotypes in adults.

Heteroresistance phenotypes were respectively observed for 83.3% (15/18) and 88.9% (16/18) of the tested antibiotics in pediatric and adult CPE-BSIs isolates. In children, the proportion of isolates with heteroresistance ranged from 93.8% for FOS to 2.1% for CT, while in adults, it was 74.0% for FOS and 1.3% for TE (Table 3). Furthermore, multiple-heteroresistance phenotypes were frequently observed in both populations (Figure 4).

**Table 3:** The frequencies of heteroresistance phenotypes to different antibiotics in both populations.

**Antibiotics**	**Appearance of heteroresistant phenotypes in children samples** ***N* (%),** ***n* = 48**	**Appearance of heteroresistant phenotypes in adult samples** ***N* (%),** ***n* = 77**
Amikacin	17 (35.4%)	4 (5.2%)
Tobramycin	12 (25.0%)	10 (13.0%)
Ciprofloxacin	5 (10.4%)	9 (11.7%)
Minocycline	13 (27.1%)	10 (13.0%)
Fosfomycin	45 (93.8%)	57 (74.0%)
Ceftriaxone	0 (0.0%)	6 (7.8%)
Aztreonam	7 (14.6%)	6 (7.8%)
Tetracycline	7 (14.6%)	1 (1.3%)
Polymyxin B	0 (0.0%)	0 (0.0%)
Ertapenem	7 (14.6%)	19 (24.7%)
Imipenem	28 (58.3%)	30 (39.0%)
Meropenem	19 (39.6%)	24 (31.2%)
Ceftazidime	0 (0.0%)	11 (14.3%)
Ceftazidime/Avibactam	8 (16.7%)	3 (3.9%)
Cefepime	15 (31.3%)	20 (26.0%)
Piperacillin/Tazobactam	3 (6.3%)	14 (18.2%)
Tigecycline	6 (12.5%)	3 (3.9%)
Colistin	1 (2.1%)	0 (0.0%)

*Note: The color denotes that the larger the value is, the brighter the color is*.

The proportion of different species exhibiting heteroresistance to different drug combinations was various. Surprisingly, Table 4 shows that some common multiple-heteroresistance phenotypes cover a significant percentage of some species. Different species were given specific combination recipes based on their multiple-heteroresistance phenotype.

**Table 4:** The frequencies of common multiple-heteroresistance combinations in isolates of different species from children.

**Populations and species**	**Multiple heteroresistance combination**	**Frequency (rate)**
**Children (*n* = 48)**		
***E. coli*** **(*n* = 18)**	**FOS+MH**	**7 (39%)**
	FOS+AK	6 (33%)
	FOS+IPM	4 (22%)
***K. pneumoniae*** **(*n* = 22)**	**FOS+IPM**	**16 (73%)**
	FOS+FEP	10 (45%)
	FOS+MEM	10 (45%)
***E. cloacae*** **(*n* = 8)**	**FOS+AK+IPM+MEM**	**8 (100%)**
**Adults (*n* = 77)**		
***E. coli*** **(*n* = 10)**	FOS+FEP	**2 (20%)**
	FOS+TZP	2 (20%)
	FOS+IPM	2 (20%)
***K. pneumoniae*** **(*n* = 49)**	**FOS+IPM**	**10 (20%)**
	FOS+CAZ	7 (14%)
	IPM+CAZ	6 (12%)
***E. cloacae*** **(*n* = 5)**	**FOS+IPM/TZP**	**4 (80%)**
	FOS+MEM/ETP	**3 (60%)**
	FOS+FEP	**2 (40%)**
***C. freudii*** **(*n* = 12)**	**IPM+FEP**	**11 (92%)**
	IPM+FOS/MEM	10 (83%)
	FOS+FEP	9 (75%)

*77 of 78 stains from adults were tested, and the rest one was unavailable. Note: The bold value denotes the most common combination in different species. The color denotes different species*.

**Figure 5:**
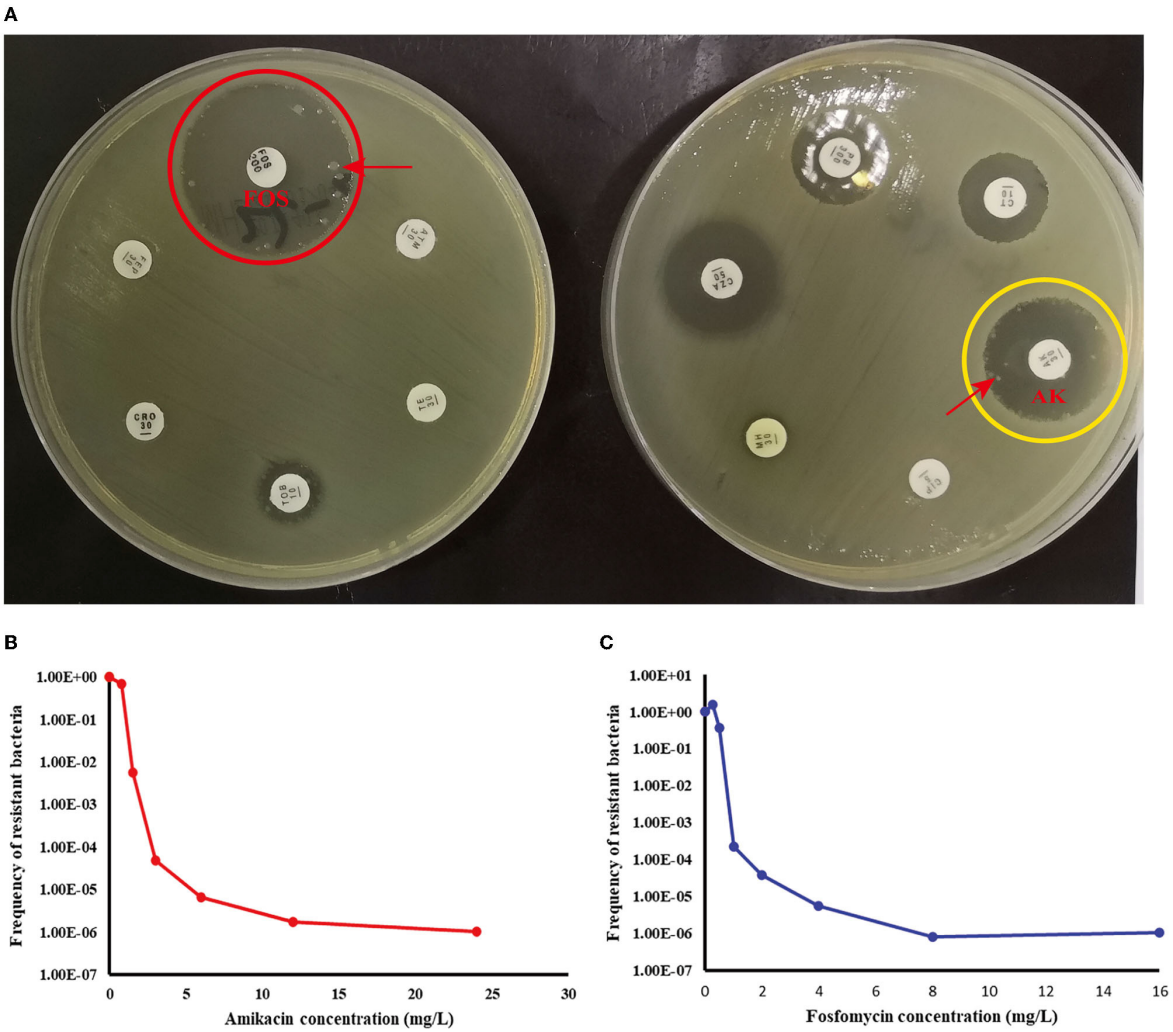
Children #22 strain (*E. coli*) showed FOS- and AK-co-heteroresistance. **(A)** K-B tests: the heteroresistance phenotypes of the strain to the two drugs. **(B)** The PAP curve of amikacin. **(C)** The PAP curve of fosfomycin. Each value was the average of the results of three repeated experiments. The highest drug concentrations on the plates (FOS and AK) were 4 times the MIC for each drug, and the heteroresistant frequencies of both drugs were >1 × 10^−7^. Red arrows indicated heteroresistant colonies.

**Figure 6:**
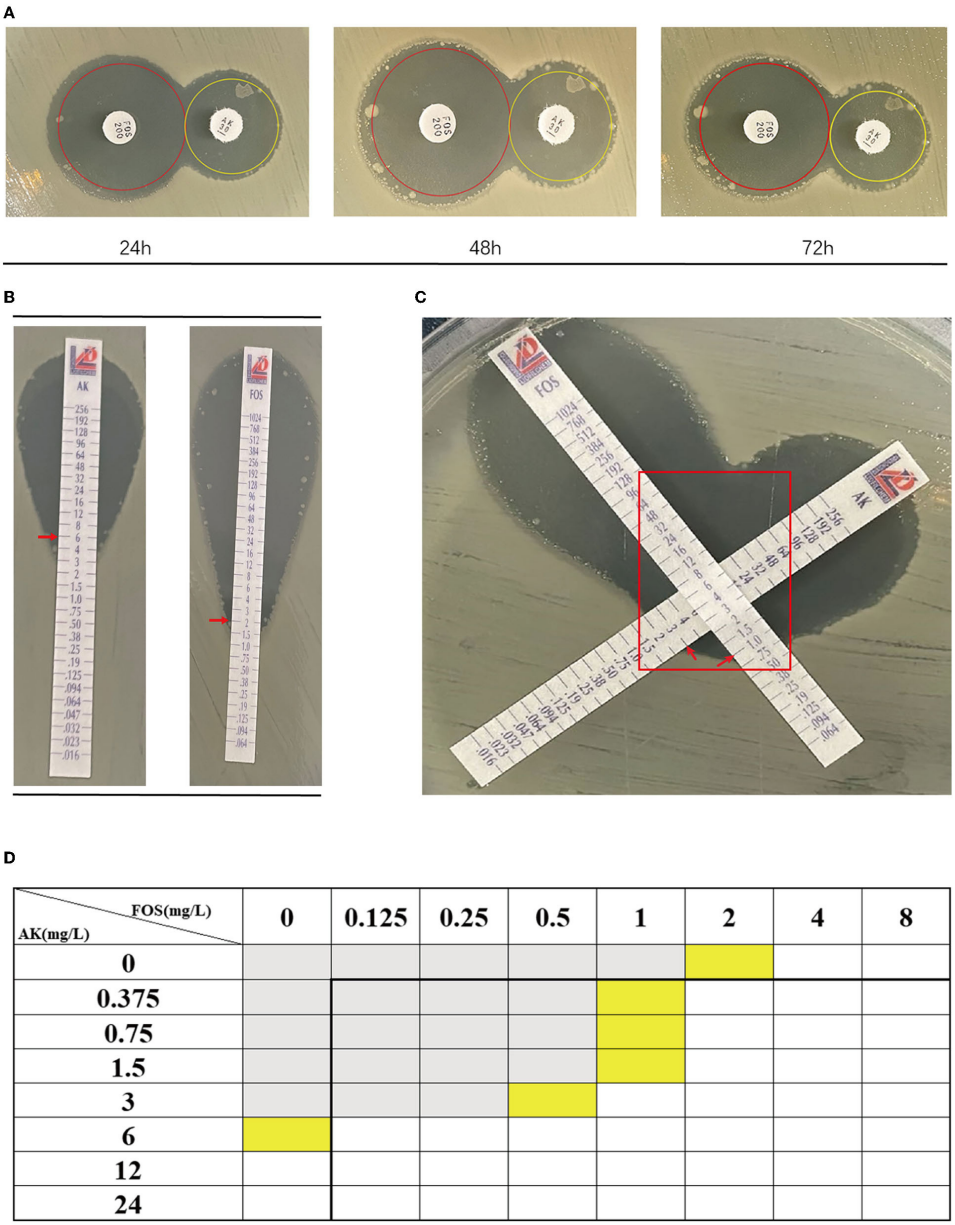
The combination of FOS and AK showed an additive effect on Children #22 strain. **(A)** Dual K-B tests. The results showed that the effect of the combination of FOS and AK on the Children #22 strain was additive. The pictures from right to left were taken at 24, 48, and 72 h, respectively. There was no colony at the interface of the two antibiotic disks. **(B)** The E-test results of Children #22 strain to AK and FOS. Red rectangle: there was no colony at the interface of the two antibiotic strips. **(C)** The modified E-test showed that the combination of FOS and AK on Children #22 strain was additive (FICI = 1) according to the interpretation by Liofilchem. Red arrows on the pictures point to MICs. **(D)** A schematic of the checkboard assay: the FICI of the assay was 0.75, indicating additive effect. Gray grids showed that bacteria were growing, yellow grids indicated MIC, and white grids showed that no bacteria were growing.

According to Table 4, a typical pediatric *E coli* strain, #22 strain, was demonstrated to be heteroresistant to both FOS and AK by dual K-B tests and gold standard PAP (Figure 5). We further evaluated the antimicrobial effect of the combination therapy with FOS and AK on the FOS- and AK-co-heteroresistant strain. In dual K-B tests, there was no colony growing at the interface of the two disks, showing a potential additive effect (Figure 6A), which was further supported by E-tests result with a FICI of 1 (Figures 6B,C). We finally used agar dilution checkboard assay, the gold standard method for synergy testing. As expected, the FICI (0.75) of the gold standard further indicated that the effect of the combination was additive (Figure 6D).

## Discussion

Antibiotic treatment for CPE-BSIs is concerning, and the spread of resistance cannot be determined solely based on plasmids or chromosomal lineages (26). Our work comprehensively compared both the clinical and microbiological characteristics of CPE-BSIs in children and adults. We also explored the possibility of designing a combination therapy strategy based on multiple-heteroresistance phenotypes. The research not only filled a knowledge gap in children with CPE-BSIs, but it also discovered the principle behind resistance spread and an effective way to design antibiotic combination therapy.

From the perspective of the clinic, children had a significantly lower 30-day crude mortality when compared with that of adults. We further analyzed the antibiotics administered in both populations. 45.8% of the children were given amikacin as an active drug on an empirical basis, and there was no difference in mortality between those who received amikacin and those who did not. Although aminoglycoside is not recommended in children for its ototoxicity and nephrotoxicity (27), amikacin might be outstanding for its excellent bactericidal activity, thus being the choice of the clinicians in cases where no other effective antibiotics were available. While 38.4 percent of adults were given tigecycline as an active drug on an empirical basis, the tigecycline-treated subgroup had significantly higher 30-day crude mortality. Tigecycline is recommended for CPE infections by many researchers (28–30). In Chinese consensus (29), most combination therapies to XDR *Enterobacteriaceae* are based on tigecycline, and researches showed that the combination including tigecycline was active to CRE infections, which was contrary to our results. It may be due to 3 reasons: (1) the subject of our study was confined to CPE; (2) severe side-effect of tigecycline; (3) we did not consider confounders, such as the severity of the illness. Anyway, our results indicated that the utility of tigecycline for CPE infections should be reconsidered and limited, rather than taking it as a panacea. However, depending on the underlying conditions and microbiological characteristics, amikacin may be suitable for neonates regardless of toxicity. Notably, nosocomial CPE-BSIs accounted respectively for 81.3% (39/48) and 78.2% (61/78) in the pediatric and adult populations. Some of the explanations for the higher distribution of CPE in nosocomial settings might be the promoted clonal spread and horizontal gene transfer under potent antimicrobial pressure in the hospital environment. CPE transmission in nosocomial settings may also have been aided by frequent invasive procedures and a lack of control measures in healthcare settings aimed at CPE carriers. From the view of microbiology, *E. coli* isolates tended to carry *bla*_NDM_, especially *bla*_NDM−5_, whilst *E. cloacae* strains tended to express MBLs, especially NDM-1, no matter in children or adults. Diverse STs in all species tested tended to carry *bla*_NDM_ without preferences. The low fitness cost of *bla*_NDM_ may account for the phenomena, regardless of the plasmids or clonal lineages. Carolina and her colleagues have proved that NDM is of a unique structure and located at the membrane, and these features confer its lowest fitness cost among a series of classical MBLs (8, 31). Furthermore, while it has been reported that plasmids carrying *bla*_NDM_ often co-harbor the resistance genes to aminoglycosides (32, 33), we discovered that the majority of *bla*_NDM_-positive isolates from children were amikacin-susceptible.

Consistent with some previous studies (34, 35), ST11 *bla*_KPC−2_-producing *K. pneumoniae* (42.3%, 33/78) was the mainstay in adults, while in some other previous studies, the successful clonal lineage of *bla*_KPC_ is ST258/512 (26). Interestingly, ST258 was recognized as a product of the recombination event between ST11 and ST442 (36). There may be some advantages of these STs that contribute to transmission. However, this cannot explain the predominance of *bla*_NDM_-producing *K. pneumoniae* in children. Host immunity may be one of the determinants that affect the strategy that *K. pneumoniae* is taken to attack, since in our study, *E. coli* seems to be more readily to infect children instead of adults and the immunity of both populations is of great difference (37).

Because of their efficient carbapenemase activity against practically all β-lactam antibiotics except ATM, potent horizontal transfer, and lack of therapeutic inhibitors, the spread of NDM in pediatric CPE-BSI isolates should be regarded as the most urgent concern. Therapy of CPE-BSIs due to NDM-producing *Enterobacterales* is extremely challenging, as most *bla*_NDM_-encoding plasmids co-harbored multiple resistance determinants, including serine-β-lactamase (SBLs) for ATM, 16S RNA methylases for aminoglycosides, rifampin-modifying enzymes for rifampin, chloramphenicol acetyltransferase for chloramphenicol, and esterase for macrolides, thus expanding to multi-drug resistance (MDR), extreme-drug resistance (XDR), and pan-drug resistance (PDR) (38). Avibactam (AVI) is a non-β-lactam-β-lactamase inhibitor which can inhibit most SBLs, including class A (KPC), class C (AmpC), and certain class D (OXA-48) enzymes, but can neither inhibit MBLs nor protect β-lactams from MBLs hydrolysis (39). The addition of AVI to ATM can theoretically protect ATM from the hydrolysis by SBLs and therefore can expand the spectrum of ATM to cover *Enterobacterales* with class A, B, C, and some D β-lactamases. As expected, the ATM-AVI combination showed robust *in vitro* action against KPC producers, NDM-positive isolates, and even KPC and NDM co-producers in our study, with a susceptible rate of 100.0% in pediatric isolates and 98.7% in adult strains, respectively (Table 2). Nevertheless, the possible clinical development of ATM-AVI resistance is of particular concern. In fact, decreased susceptibilities and resistance to ATM-AVI have been recently witnessed in clinical *E. coli* strains from India (40), China (41), Europe, Africa, Asia, and Australia (42), due to PBP3 insertions and CMY-42 production. Of note, our present study has also demonstrated decreased susceptibilities to ATM-AVI (MIC = 8 mg/L) in one NDM-5-producing adult *E. coli* isolate from the First Affiliated Hospital of Chongqing Medical University (Table 2). The observation that PBP3 insertions and CMY-42 production in *E. coli* could adversely impact ATM-AVI activity against MBLs-producing *Enterobacteriaceae* (MPE) reveals a clinically significant spectrum gap in this MBL-targeted therapy. Such vulnerability of ATM-AVI could also compromise the combined effectiveness of CAZ-AVI and ATM (both targeting PBP3), being viewed as a “rescue therapy” for serious MBL-producer infections (43, 44).

CAZ-AVI, on the other hand, has provided opportunities for safe and effective therapies for serious infections caused by some of the most threatening pathogens producing ESBLs, KPC, AmpC, and OXA-48, but it showed no *in vitro* activity against MPE (45), as evidenced by our AST results, which showed a low CAZ-AVI-susceptible rate of 14.6% in pediatric isolates (most of them are MPE) and a much higher 62.3% susceptible rate in the adult isolates (most of them are KPC-producers) (Table 2).

Multiple-heteroresistance may help to improve the efficacy of antimicrobial combination therapy design. Traditionally, combination therapy was decided on the basis of experience rather than principles, and it lacked pertinence. We strive to use multiple-heteroresistance phenotypes to tackle the problems: combining the antibiotics showing multiple-heteroresistance phenotypes had a synergistic effect (20), and we discovered that the multiple-heteroresistance profiles of different species were different (Table 4). We finally identified a combination of FOS and AK showing the additive effect on a FOS- and AK-co-heteroresistant *E coli* strain by this way, and such a combination has been reported recently to be of efficiency without a theoretical basis (46). There were concerns about the toxicity of the two drugs, but reducing doses of the two drugs when combined together could decrease the toxicity of each drug (27, 47, 48). Our study proved the feasibility of the way to design combination therapy based on multiple-heteroresistance, and in this way, we can not only reduce the workload in finding efficient combination but also confer the features of experimental evidence and theoretical basis to the combination therapy. Nevertheless, further investigations from more CPE isolates of various species are needed to evaluate the possible *in vitro* synergy of the other antimicrobial combinations based on the multiple-heteroresistance phenotypes. Furthermore, because most *in vitro* test methods are unable to predict clinical success rates, prospective clinical studies based on *in vitro* synergy testing results are required to improve clinical outcomes.

There is only one limitation of this study is that it's a retrospective study with a relatively small sample size, and a larger prospective cohort study is still needed. Nevertheless, as the test level was kept constant (α = 0.05), and the test efficiency of our study can reach ≥80% (1-β ≥ 80%), the indicators with detected significant differences in our study were authentic.

Overall, we discovered much more detailed characteristics about the dissemination of CPE in different host populations by comparing children and adults with CPE-BSIs, and we proved that the multiple-heteroresistance profile in different species exhibited some specificity, which helped us design combination therapy.

## Data Availability Statement

The original contributions presented in the study are included in the article/supplementary material, further inquiries can be directed to the corresponding author/s.

## Ethics Statement

The study was approved by the Institutional Ethics Committee of the First Affiliated Hospital of Chongqing Medical University (Number: 2021-524).

## Author Contributions

SH, QZ, and SN designed the study. HY, DM, BL, QL, and RY performed the experimental work. DM, BL, and SY collected the data. HY and XJ analyzed the data. HY and SH wrote the article. All authors read and approved the final article.

## Funding

This work was sponsored by the National Natural Science Foundation of China (Grant Nos. 82072346 and 82072349), and the Natural Science Foundation of Chongqing, China (No. cstc2020jcyj-msxmX0519).

## Conflict of Interest

The authors declare that the research was conducted in the absence of any commercial or financial relationships that could be construed as a potential conflict of interest.

## Publisher's Note

All claims expressed in this article are solely those of the authors and do not necessarily represent those of their affiliated organizations, or those of the publisher, the editors and the reviewers. Any product that may be evaluated in this article, or claim that may be made by its manufacturer, is not guaranteed or endorsed by the publisher.
